# Visualizing the internal structural heterogeneity of *Paeonia lactiflora* starch granules by synchrotron soft X-ray microscopy

**DOI:** 10.1016/j.mex.2026.104022

**Published:** 2026-06-30

**Authors:** Jinyu Cai, Minghui Sun, Xinge Cao, Zongran Lu, Hamizah Shahirah Hamezah, Menghui He, Lan Han, Rongchun Han

**Affiliations:** a School of Pharmacy, Anhui University of Chinese Medicine, Hefei, 230012, China; b Institute of Systems Biology, Universiti Kebangsaan Malaysia, Bangi, 43600, Malaysia; c National Synchrotron Radiation Laboratory, University of Science and Technology of China, Hefei, 230029, China

**Keywords:** Soft X-ray microscopy, Dual-energy imaging, Quality assessment, *Paeonia lactiflora*, Starch granules

## Abstract

The traditional assessment of harvest timing for *Paeonia lactiflora* roots relies on the empirical “sufficient powderiness” phenotype, which lacks objective biomarkers. This protocol describes a synchrotron-based soft X-ray microscopy method at the oxygen K-edge for label-free, high-resolution visualization of oxygen distribution patterns within starch granules.

• Dual-energy imaging (530 eV and 545 eV) combined with resin embedding and 500 nm sectioning enables visualization of the characteristic oxygen-deficient core and oxygen-enriched periphery.
• ImageJ-based gray-value analysis provides relative quantitative comparison of oxygen density between optimal and non-optimal harvest stages.
• The method offers a reproducible, objective tool for harvest-time evaluation and starch ultrastructure studies in medicinal plants.

## Graphical abstract

**Figure:**
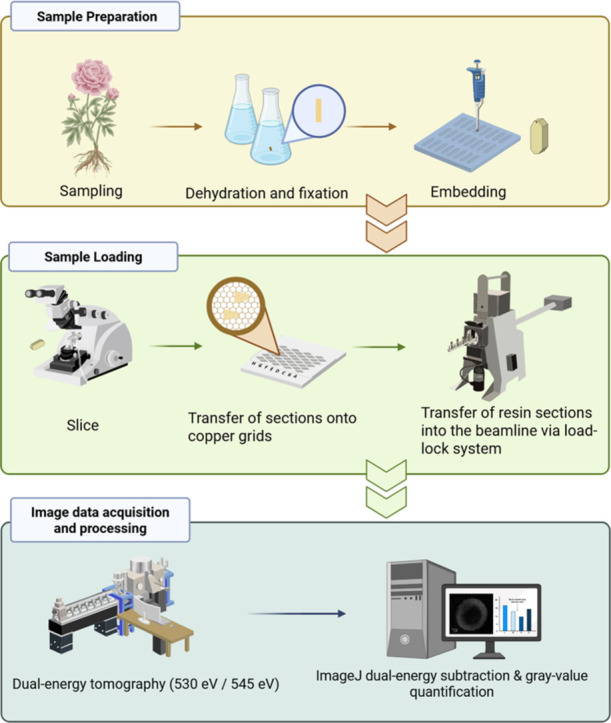

## Specifications table

**Table:** 

**Subject area**	Agricultural and Biological Sciences
**More specific subject area**	Plant science; Medicinal plant quality assessment; Synchrotron imaging
**Name of your method**	Synchrotron soft X-ray dual-energy microscopy for oxygen distribution in starch granules
**Name and reference of original method**	None
**Resource availability**	Beamline BL07W at National Synchrotron Radiation Laboratory (Hefei, China); Leica EM UC7 ultramicrotome; ImageJ (https://imagej.net/ij/); GraphPad Prism 9.0 (https://www.graphpad.com/features)

## Background

The optimal harvest time of the medicinal herb *Paeonia lactiflora* Pall. is traditionally determined by the empirical phenotype of “sufficient powderiness,” in which a fresh root cross-section appears white to pinkish with a distinctly powdery texture [1]. However, this macroscopic indicator lacks objective, quantifiable biomarkers, severely limiting standardization and reproducibility in herbal medicine quality control. Although conventional techniques—including scanning electron microscopy, X-ray diffraction, Fourier transform infrared spectroscopy, and transmission electron microscopy with energy-dispersive X-ray spectroscopy—can provide morphological, crystalline, or semi-quantitative elemental data, they fail to deliver high-resolution, label-free, in situ spatial mapping and quantitative analysis of oxygen distribution inside starch granules [[2], [3], [4], [5]].

Here we present a complete, reproducible protocol for high-resolution mapping of oxygen distribution in *P. lactiflora* root starch granules using synchrotron-based soft X-ray microscopy at the oxygen K-edge (dual-energy imaging at 530 eV pre-edge and 545 eV post-edge). The energy pair was selected based on the well-established oxygen K-edge position (∼543 eV) in organic and polysaccharide-rich materials, where 530 eV provides primarily mass-thickness reference contrast and 545 eV captures strong oxygen-specific absorption. This approach enables direct visualization of the characteristic “oxygen-deficient core and oxygen-enriched periphery” configuration in optimally harvested granules. It further permits relative quantitative comparison through ImageJ-based mean gray value analysis of dual-energy subtraction images (545 eV – 530 eV), which serves as a reliable proxy for oxygen density distribution.

In our companion study [1], this characteristic oxygen-deficient core surrounded by an oxygen-enriched periphery was shown to correlate strongly with the traditional “sufficient powderiness” phenotype: optimally harvested roots exhibit higher overall relative oxygen density and a clearer core-periphery gradient, which likely contributes to the powdery texture observed upon fracturing due to differences in starch granule compactness and hydration properties.

This protocol is an extended and fully detailed version of the methods described in our previously published original research article [1], which was necessarily abbreviated due to journal space limitations. It provides comprehensive, step-by-step instructions covering resin embedding and sectioning, beamline operation at BL07W, data acquisition, and ImageJ-based relative quantitative analysis.

The protocol is readily adaptable to other starch-rich medicinal plant roots, tubers, or seeds and offers a robust technical platform for objective harvest-time determination, quality standardization, and ultrastructural studies of starch granule development in medicinal plants.

## Method details

This protocol enables other researchers to fully replicate the synchrotron-based soft X-ray microscopy workflow for visualizing oxygen distribution patterns in medicinal plant starch granules. All steps are described in detail below.

◆ **TIMING** The complete protocol requires approximately 3–5 days (1 day for sampling, 1–2 days for embedding and sectioning, and 1–2 days for imaging and data processing), depending on sample number and beamline availability.

### Sampling

◆ **TIMING ∼2–4 h**

Fresh *P. lactiflora* roots are collected during the optimal harvest window (first week of October 2024) and non-optimal windows (first week of June and March 2024), with three independent biological replicates per stage. Medicinal root segments (1–2 cm long, 1–2 cm diameter) are excised from the middle portion of the root and immediately rinsed with ice-cold water to remove adhering soil. The segments are wrapped in moist gauze, sealed in an ice box, and transported to the laboratory while maintained at 0–4 °C to minimize oxidation and starch degradation. Upon arrival, samples are immediately snap-frozen in liquid nitrogen or stored at −80 °C.

◆ **CRITICAL:** Samples must be frozen immediately after collection to inactivate amylases and preserve native starch granule structure.
◆ **TROUBLESHOOTING:** Prolonged transportation → snap-freeze samples in liquid nitrogen directly at the collection site.

### Embedding & sectioning (resin embedding and sectioning)

• **TIMING ∼36–72 h**

**Fig. 1:**
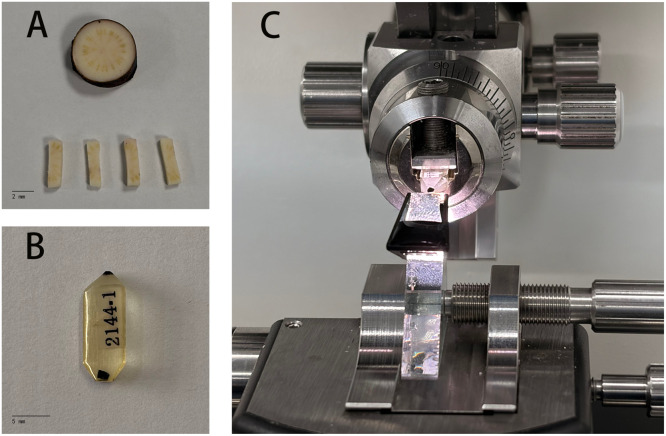
Resin embedding and sectioning procedure for soft X-ray imaging.

Fresh root segments are cut into blocks smaller than 1 mm³ (Fig. 1A) and immediately fixed in 2.5% glutaraldehyde containing 1–2% osmium tetroxide. Vacuum infiltration is applied to ensure complete penetration, followed by fixation overnight at 4 °C. Samples are dehydrated in a graded ethanol series (30% to 100%, 15–30 min per step) and infiltrated twice with propylene oxide (30 min each). Gradual infiltration with epoxy resin (Epon 812 or Spurr) is performed using acetone: resin ratios progressing from 3:1 to 1:1 to 1:3 to pure resin (4–8 h per step). Infiltrated samples are transferred to embedding molds and polymerized at 45 °C for 12 h, followed by 60 °C (or 70 °C for Spurr resin) for 24–72 h to produce hard resin blocks [6]. Blocks are trimmed into a pyramidal shape with a small cutting face to expose the tissue (Fig. 1B). Semi-thin sections (500 nm thick) are cut on a Leica EM UC7 ultramicrotome using a glass knife. The section thickness is set to 500 nm and the cutting speed is maintained at 1.0 mm s⁻¹ with a knife angle of 6° Sections are floated on water, flattened, picked up with an eyelash probe, and transferred onto copper grids supported by a carbon film.

◆ **CRITICAL:** Polymerized resin blocks may exhibit uneven hardness or residual stress. Brief heating (10–15 min at 60–70 °C) immediately before sectioning softens the resin, minimizing chatter marks, tearing, and compression artifacts.
◆ **CRITICAL:** If the sections are too thick (>1 µm), soft X-rays (particularly in the water window region) cannot effectively penetrate the sample, resulting in insufficient transmission, extremely low signal-to-noise ratio, and poor image quality. Conversely, sections that are too thin (<300 nm) may lead to insufficient structural information and increased risk of mechanical damage during handling.
◆ **TROUBLESHOOTING:** Section breakage or knife marks → reduce feed rate, re-heat the block, or replace the glass knife; uneven thickness → inspect knife edge quality and re-trim the block.

(A) Fresh root segments (<1 mm³). (B) Trimmed pyramidal resin block ready for sectioning. (C) Leica EM UC7 ultramicrotome equipped with a glass knife used to cut 500 nm semi-thin sections.

### Sample loading

• **TIMING ∼0.5 h**

**Fig. 2:**
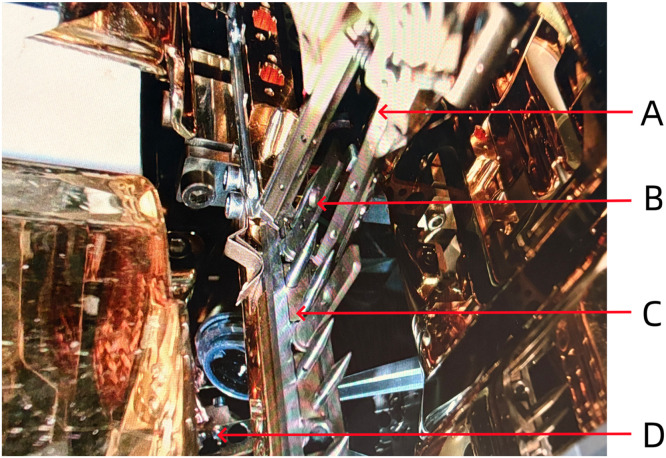
Interior view of the sample chamber at beamline BL07W.

Resin-embedded sections on copper grids are mounted onto the sample holder. The holder is transferred into the main vacuum chamber via the load-lock transfer box. Using the ZEISS XMC Controller software, the illumination source and camera are activated. The zone plate and capillary condenser are observed and retracted from the sample chamber after confirming that the Zone Plate Z position is <0. The mechanical arm (Fig. 2A) transfers the sample holder (Fig. 2B) from the internal sample shuttle (Fig. 2C) to the sample stage (Fig. 2D). The X-ray source valve is opened, the detector temperature is set to −65 °C, and the system is prepared for imaging [7].

(A) Mechanical arm for sample transfer. (B) Sample holder with mounted copper grid. (C) Internal sample shuttle. (D) Sample stage.

### Imaging operation and data acquisition

• **TIMING ∼4–8** **h** (depending on sample number)

**Fig. 3:**
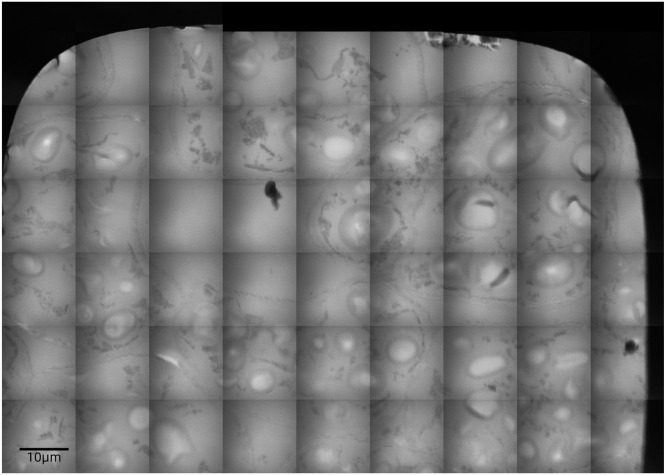
Representative Mosaic scanning image during data acquisition. Mosaic mode is used to locate starch granules within the 500 nm resin section on the copper grid. The target granule is identified and moved to the center of the field of view for subsequent dual-energy 2D imaging.

The sample stage is rotated to Sample Theta = −60° An optimal field of view containing starch granules is located on the copper grid using the visible-light microscope (VLM) and centered. The stage is rotated to Theta = 0°, and coarse focusing is performed with the PIXIS CCD detector in continuous acquisition mode. The system is switched to Mosaic mode to scan for starch granule structures (Fig. 3). Once located, the target granule is precisely centered and finely focused.

Dual-energy 2D projection images are acquired at 530 eV (pre-edge) and 545 eV (post-edge) using Tomography mode (which in this context is employed for automated 2D scanning and projection acquisition rather than full 3D tomography, given the 500 nm thin sections). User-defined start and end angles are set according to the grid geometry to avoid shading. During energy switching, the Zone Plate Z offset is recalculated using beamline-specific focus calibration curves. The adjustment compensates for the photon-energy dependence of the zone plate focal length (f ∝ E), which causes the focal plane to shift when the X-ray energy changes. The required correction therefore depends on the calibrated relationship among photon energy, focal length, and Zone Plate Z position. Users operating at facilities other than BL07W should consult local beamline documentation or scientists for the corresponding calibration parameters [8]. Exposure time is iteratively optimized (initial range 1–5 s, adjusted in real time according to beam current) to ensure the maximum gray value reaches 80–90% of the detector dynamic range. After acquisition, the sample holder is moved out of the beam path or a clean region is selected, and background images are collected using Averaging 10 mode [2]. All samples are recovered undamaged at the end of the session.

◆ **CRITICAL:** The energies 530 eV and 545 eV were chosen respectively as below- and above-edge energies with reference to the well-documented O K-edge position at ∼543.1 eV [8], as well as the favorable photon flux within the water window. The directly measured O K edge X-ray Absorption Near Edge Structure (XANES) of cassava starch reported by Julia [9], which confirms that 530 eV lies in a pre-edge region of negligible oxygen absorption. This selection ensures sensitive and consistent oxygen contrast, as verified by extensive O K-edge XANES studies on inorganic matter [10,11].
◆ **CRITICAL:** Exposure time directly controls photon accumulation (typical flux ∼2 × 10¹⁰ photons/s in the water window). Too short an exposure yields insufficient signal-to-noise ratio and poor reconstruction accuracy; excessively long exposures cause radiation damage and reduce throughput. The ideal histogram peak lies at 80–90% of detector dynamic range.
◆ **CRITICAL:** Beamline BL07W is equipped with an ellipsoidal capillary condenser and a Zeiss Ni zone plate (25 nm outermost zone width), delivering a spatial resolution of ∼30 nm.
◆ **TROUBLESHOOTING:** Image too dark (low SNR) → increase exposure time by 1–2 s increments; image saturated (large overexposed areas) → decrease exposure time by 0.5–1 s increments; missing projections → adjust rotation range to avoid grid shading [12].

### Data processing

• **TIMING ∼4–8 h**

Raw 2D projection images are imported into ImageJ and background is subtracted (Process → Subtract Background). The 530 eV and 545 eV image stacks are aligned using pixel registration (Plugins → Registration → Register Virtual Stack Slices or TurboReg plugin, ensuring sub-pixel accuracy) [13]. Dual-energy subtraction is performed (545 eV − 530 eV) to generate oxygen-specific contrast images.

The resulting images are converted to 8-bit grayscale. Individual starch granules are manually selected as regions of interest (ROI) using the freehand or wand tool, carefully excluding section edges and artifacts. Mean gray values are measured for each ROI (Analyze → Measure); these values serve as a proxy for relative oxygen density [14]. Data are exported to GraphPad Prism 9.0 for statistical analysis. Results are presented as mean ± SEM. Statistical significance among harvest stages was evaluated using one-way ANOVA followed by Tukey’s multiple comparisons test. Differences were considered statistically significant at p < 0.01.

◆ CRITICAL: At least three independent starch granules must be analyzed per sample. Identical threshold settings must be applied to all samples within a batch to ensure comparability.
◆ **TROUBLESHOOTING:** Large gray-value fluctuations → verify registration accuracy or repeat background subtraction; prominent artifacts → confirm correct Zone Plate Z offset during energy switching.

## Method validation

Application of this protocol to samples from optimal and non-optimal harvest stages consistently reveals a distinct structural heterogeneity: an oxygen-deficient core surrounded by an oxygen-enriched periphery. This characteristic pattern is prominently observed in optimally harvested granules, with mean gray values significantly higher than those from non-optimal stages (n = 3 biological replicates, one-way ANOVA with Tukey’s multiple comparisons test, p < 0.01).

Control experiments, including imaging of resin-only regions and non-starch cellular components, confirm that the observed oxygen distribution is an intrinsic structural feature of the starch granules rather than an artifact of sample preparation or imaging conditions. Furthermore, the results obtained with the present protocol align well with preliminary TEM-EDS observations but offer markedly superior spatial resolution (∼30 nm), enabling clearer visualization of the core-periphery oxygen gradient and finer ultrastructural details within individual granules [1].

## Limitations

1. Requires access to a synchrotron soft X-ray beamline (e.g., BL07W), which involves competitive beamtime allocation and is not available in standard laboratories.
2. High-quality 500 nm resin sectioning demands specialized ultramicrotomy skills and equipment. Sections that are too thick will prevent adequate soft X-ray penetration, while excessively thin sections compromise mechanical stability and contrast.
3. The dual-energy subtraction provides relative rather than absolute oxygen density mapping. Absolute quantification would require full XANES spectral stacks and appropriate reference standards.
4. The method is destructive (sectioning required) and not suitable for real-time or in vivo imaging of living tissues.
5. Prolonged X-ray exposure may cause minor radiation damage to delicate biological structures.

## Ethics statements

Not applicable (plant material only; no human or animal subjects involved).

## Related research article

Lu, Z., Cheng, Y., Cai, J., Han, L., He, M., … Han, R. Starch granules of distinct harvest time under soft X-ray microscopy: From empirical sufficient powderiness to oxygen density. Ind. Crops Prod. 243 (2026) 123,115. https://doi.org/10.1016/j.indcrop.2026.123115

## Declaration of competing interest

The authors declare that they have no known competing financial interests or personal relationships that could have appeared to influence the work reported in this paper.

## Data Availability

No data was used for the research described in the article.
